# Role of Knowledge Management on the Sustainable Environment: Assessing the Moderating Effect of Innovative Culture

**DOI:** 10.3389/fpsyg.2022.861813

**Published:** 2022-04-07

**Authors:** An Weina, Yang Yanling

**Affiliations:** ^1^College of Health Management, Xian Medical University, Xi'an, China; ^2^Institute of Culture and History, Shaanxi Academy of Social Sciences, Xi'an, China

**Keywords:** knowledge management practices, sustainable environment, environment awareness, green technological use, green innovative culture

## Abstract

Environmental sustainability has become the need of the hour and has been emphasized immensely because of the increased environmental awareness and resulting problems caused due to negligence. This study has intended to determine the role of knowledge management (KM) practices in achieving a sustainable environment with the mediating role of environmental awareness and green technological use. The study further examined the moderating role of green innovative culture between the relationship of KM practices and a sustainable environment. The data were acquired from 378 managerial level personnel of the construction industry in China through questionnaires. Smart-PLS 3.3.3 was used to determine the study's hypothesis through the structural equation modeling (SEM) technique. The study found that KM practice has a significant relationship with a sustainable environment, environmental awareness, and green technological use. Also, environmental awareness has a significant effect on a sustainable environment. Moreover, it was found in the study that environmental awareness significantly mediated the relationship between KM practices and sustainable environment, but green technological use did not find any mediating effect on the relationship between KM practices and sustainable environment. Furthermore, green innovative culture considerably moderated the relationship between KM practices and a sustainable environment. Theoretically, this study contributes to the existing literature by incorporating and investigating the role of KM practices in a sustainable environment. Practically, this article presented some implications for the management concerning promoting KM practices and environmental awareness within the organization and developing a green innovative culture.

## Introduction

The United Nations Agenda 2030, and the 17 Sustainable Development Goals in general, have given a new stimulus to the consequences of sustainable development, which is defined as a process leading to the more rational use of natural resources based on the principles of environmental equity, as well as social equity for the resolutions of this object (Palomares et al., 2021). Sustainability has increased in accordance or significance for firms seeking a competitive edge or uniqueness point, in addition to its development in other areas of society (Streimikiene et al., 2021). Knowledge Management (KM) might be useful in this situation. The reason for this is that KM has grown in importance as a means of ensuring and maintaining competitive advantages for businesses (Fu et al., 2022). The desire to look for, absorb, and share knowledge has made a significant contribution to the accomplishment of corporate objectives (Olabi et al., 2022). For enterprises of all areas and expertise, KM is seen as a critical strategic resource (Gloet and Samson, 2022). It is imperative to remember that, due to its own invulnerability, information is difficult to comprehend, communicate, as well as spread throughout an organization's many sectors. Gaining competitive advantage requires the effective and continuous use of knowledge (Jewell et al., 2022).

Investment in KM ensures that all of an organization's knowledge is put to good use (Yang et al., 2022). When KM is employed in the context of sustainability, the organization's attitude shifts, and social and environmental responsibility is given equal weight to economic viability (Ikram et al., 2021). Sustainable development techniques may be built on the foundation of KM (Frolova et al., 2021). Because of the difficulty of adhering to the sustainability criteria, such a union is critical (Ghadge et al., 2021). As a result, businesses must rely more heavily on their knowledge-generating process and resources (Mahdi et al., 2019). In the context of sustainability, KM is viewed as a new concept of development aimed at improving adherence to economic, environmental, and social sustainability principles (Martins et al., 2019). The aim of this research is to investigate how KM measurements or its aspects (such as knowledge dissemination, distribution and also responsiveness) affect green innovation (GI) (Hindrawati et al., 2022). Most importantly, an important part of GIs is knowing how to successfully manage KM process (Ikram et al., 2022). Despite this, there was a lack of research presenting the importance of KM for long-term business success (Kavalić et al., 2021).

Knowledge management is complicated and critical for gaining and achieving a competitive edge and epitomizes a substantial strategic potential for companies and enterprises that use GI (Song and Yu, 2018). There has been a focus on innovation for environmental sustainability (Fernando et al., 2019).

In recent decades, it has become progressively critical and crucial to businesses with decision-makers (Sénéchal and Trentesaux, 2019). GI was given special attention by researchers and scholars in marketing, the environment, business, and ethics (Kraus et al., 2020). Green technologies would increase environmental sustainability while also assisting enterprises in creating a competitive edge (Appolloni et al., 2022). However, in certain circumstances, despite the reputation and prospects of GI, producer participation in this sector has fallen short of the anticipations because of precise and certain concerns (Awan et al., 2021).

Green innovation strategy is not just a unique approach to achieving sustainable development, but it is still a necessary improvement option for businesses (Ogbeibu et al., 2021). Currently, most research on the factors driving GI strategy focuses on the direct influence of a single element, rather than taking into consideration the whole micro and macro environment (Ahmad et al., 2021). GI strategy is a business strategy that actively reduces the environmental effect of commercial activities while also incorporating commitment to the environment into strategy development (Liu et al., 2022). On the one side, as environmental rules improve and customers become more environmentally conscious, businesses are faced with more restraints (Schaltegger and Synnestvedt, 2002). Enterprises can only get distinctive competitive advantages by incorporating environmental issues into strategic height (Ngugi et al., 2021). On the other hand, due to the combined externalities of significant investment costs and high risk associated with environmental management, businesses have little motivation to pursue green solutions (Dar et al., 2022).

To recognize and identify the KM practice and its implications on the sustainable environment, environmental awareness, and green technology usage, there are numerous matters that need to be addressed in this research. Due to increased environmental awareness as well as the resulting difficulties created by its negligence, sustainable environment knowledge has become a requirement and has been highlighted greatly in this study. The goal of this study was to find out the relationship between KM practices on environmental awareness and green technology usage and the meditating role of green technology between KM and environmental awareness.

## Theoretical Framework and Hypotheses Development

### Knowledge Management Practice on the Sustainable Environment

Organizations all across the globe acknowledge KM as a key skill, the main source of competitive advantage, and a key value development (Castellani et al., 2022). Many authors in the literature, both in the public and commercial sectors, emphasize the importance of KM as a critical component for an organization's success (Benabdellah et al., 2021). Despite the significance of KM, many businesses are having difficulty adopting it successfully owing to cultural hurdles (Maravilhas and Martins, 2019). For academia and practitioners, the idea of KM has become an essential field of research in modern leadership and management (Abdulmuhsin et al., 2021). Researchers agree that KM is a collaborative and integrated method that enables a company to generate, capture, organize, access, and utilize intellectual assets and resources for long-term purpose and sustainability as well as for strategic advantage (Avotra et al., 2021). The significance of KM centralization at the worldwide level demonstrates the convergence in the application of KM in organizations.

Learning and knowledge production culture, organizational knowledge architecture for adaptive and exemptive capability, and business model for knowledge monetization and value capture are three processes that describe KM activities. The KM relationship in the context of sustainability is the KM theory employed in this study. The worldwide interchange of knowledge is critical for the viability of sustainable development under this approach (Zhang et al., 2022). In this regard, KM can be useful since it allows for the exchange of information from many time periods and locations (Gardeazabal et al., 2021). There is a significant need for approaches to enhance KM processes and procedures throughout the evaluation of environmental, psychosocial, and/or economic consequences, given the increased need for sustainability features.

***H1:*** *There is a relationship between KM practices on a sustainable environment*.

### Knowledge Management Practices on Environment Awareness and Green Technology Usage

Knowledge is being learned, shared, and applied in order to achieve and maintain competitive advantages and improve customer satisfaction (Li et al., 2021). KMP supports and helps firms to sustain and change operational sustainability as well as achieve a competitive advantage, resulting in shareholder as well as customer trust (Nayak et al., 2021). In today's corporate world, KMP has been acknowledged as an imperative component and aspect in creating and developing new services and products, and also handling and managing efficiently the operational process (Shahzad et al., 2021). As a result, companies endeavor and strive to espouse innovative and effective KM practices in order to accomplish long-term goals (Di Vaio et al., 2021). Modern economies are built using innovative ideas from human intellectual capital, which are contributing to sustainability and profitability (Yingfei et al., 2021). KMP assists organizations in building up the capabilities necessary for green innovation (GI), which further enhances CSP (Hussain et al., 2021). GIs are derivative from KMP and have contributed to the development of environmental and eco-friendly products (Nawaz et al., 2021).

From this, it is evident that KMP can play a fundamental role in attaining CSP. Knowledge resources and capabilities are the building blocks for firms' abilities to innovate sustainably (Abubakar et al., 2019). KM is critical to gaining a competitive edge and represents a substantial strategic potential for companies that embrace GI (Gope et al., 2018). In recent decades, corporate decision-makers have placed a greater premium on innovation as a means of ensuring a green environment (Xiaolong et al., 2021). GI was given special attention by researchers in marketing, the environment, business, and ethics (Ali G. et al., 2021). Green technologies would increase environmental sustainability while also assisting enterprises in gaining a competitive advantage (Tu and Wu, 2021). The value of KMP has long been recognized, and past research has found it to be an important aspect to consider when examining organizational performance, especially knowledge-based innovation (Shahzadi et al., 2021). Therefore, we resolved these hypotheses to analyze the relationship between KM practices on environmental awareness and green technology usage.

***H2a:*** *There is a relationship between KM practices on environmental awareness*.
***H2b:*** *There is a relationship between KM practices on green technological usage*.

### Environmental Awareness on Practice on Sustainable Environment

There is an increase in demand for environmentally friendly company operations as people become more conscious of environmental challenges and consequences (Lin and Niu, 2018). Preceding research has already predicted that recent or upcoming and potential stakeholder groups impact the implementation of environmental management practices through external pressures from legislators, environmental organizations, financial institutions, and suppliers, along with internal pressures from employees and also owner/manager attitudes and knowledge (Halkos and Nomikos, 2021). Knowledge awareness is derived from personal experience and routine acts as viewed through various media. Awareness matures as a result of one's psychological process, and it corresponds to the connected emotions and experiences about a certain action (Gu et al., 2022). Previous research has shown that being cognizant of green practices can lead to a positive and favorable attitude about contradancing them (Sadiq et al., 2022). People who really are aware of the possible consequences of non-sustainable activities such as improper disposal of plastic and other wastes, for example, might create a strong sense of self-belief and moral duty to take necessary action to reduce such practices (Ojo and Fauzi, 2020). The concept of “green technology” is a subset of green technologies that is used to safeguard the environment (Fernando et al., 2016). Green technology is an environmentally friendly technology that lowers the environmental damage caused by traditional technology products (Maniglia et al., 2021). These are the techniques that allow progress to continue.

It is thought that the application of green technology can aid in environmental healing, hence improving people's lives (Wang F. et al., 2021). Organizations are establishing strategies that seek a sustainable goal as the world progresses toward a healthier environment (Movilla-Pateiro et al., 2021). Green technology was defined as a collection of technologies that integrate techniques and equipment used in product design, production, and distribution to improve efficiency, decrease energy and water waste, and alleviate environmental issues (Cesar da Silva et al., 2021).

***H3a:*** *There is a relationship between environmental awareness on practice on sustainable environment*.
***H3b:*** *There is a relationship between green technological usages on sustainable environment*.

### Mediating Roles of Environmental Awareness and Green Technological Usage

When something concerns attitude toward environmental sustainability, a number of studies have found that consumer understanding of environmental issues influences their decision to buy and consume organic food (Ali L. et al., 2021). Sharing knowledge and raising awareness about environmental challenges and solutions is what environmental awareness entails (Simsar, 2021). Furthermore, the terms “environmental awareness” and “environmental knowledge” were used interchangeably, and the major drivers of green behavior were studied (Fu et al., 2020). The expansion of environmental consciousness and sensitivity education is critical for the overall well-being of society (Greven et al., 2019). Growing environmental awareness, along with concerns about safe foods, has prompted many to challenge current agricultural techniques in order to ensure the environment's long-term viability (Bertola et al., 2021). It is seen as a critical factor influencing individual consumption patterns and also environmental sustainability (Kumar et al., 2021).

Environmental knowledge research evaluates consumer understanding of environmental issues, attitudes, and their impact on the ecosystem (Liobikiene and Poškus, 2019). Environmental awareness is described as having a broad understanding of environmental facts, ideas, and interactions (Monroe et al., 2019). Green marketing as well as environmental concerns have become a hot topic among practitioners and academics alike, and several studies have been conducted to continue investigating the link between green marketing and company performance, with a focus on environmentally friendly practices and products, while on the other hand other scholars and researches have been published to investigate and examine the antecedents and factors or aspects that influence consumer attitudes toward green products (Papadas et al., 2019). As a result of this support from the literature, we hypothesized the following.

***H4a:*** *Environmental awareness mediates the relationship between KM practices on environment awareness*.
***H4b:*** *Green technological usage mediates the relationship between KM practices on environmental awareness*.
***H5:*** *Green Innovative culture moderates the relationship between KM and environmental awareness*.

**Figure 1:**
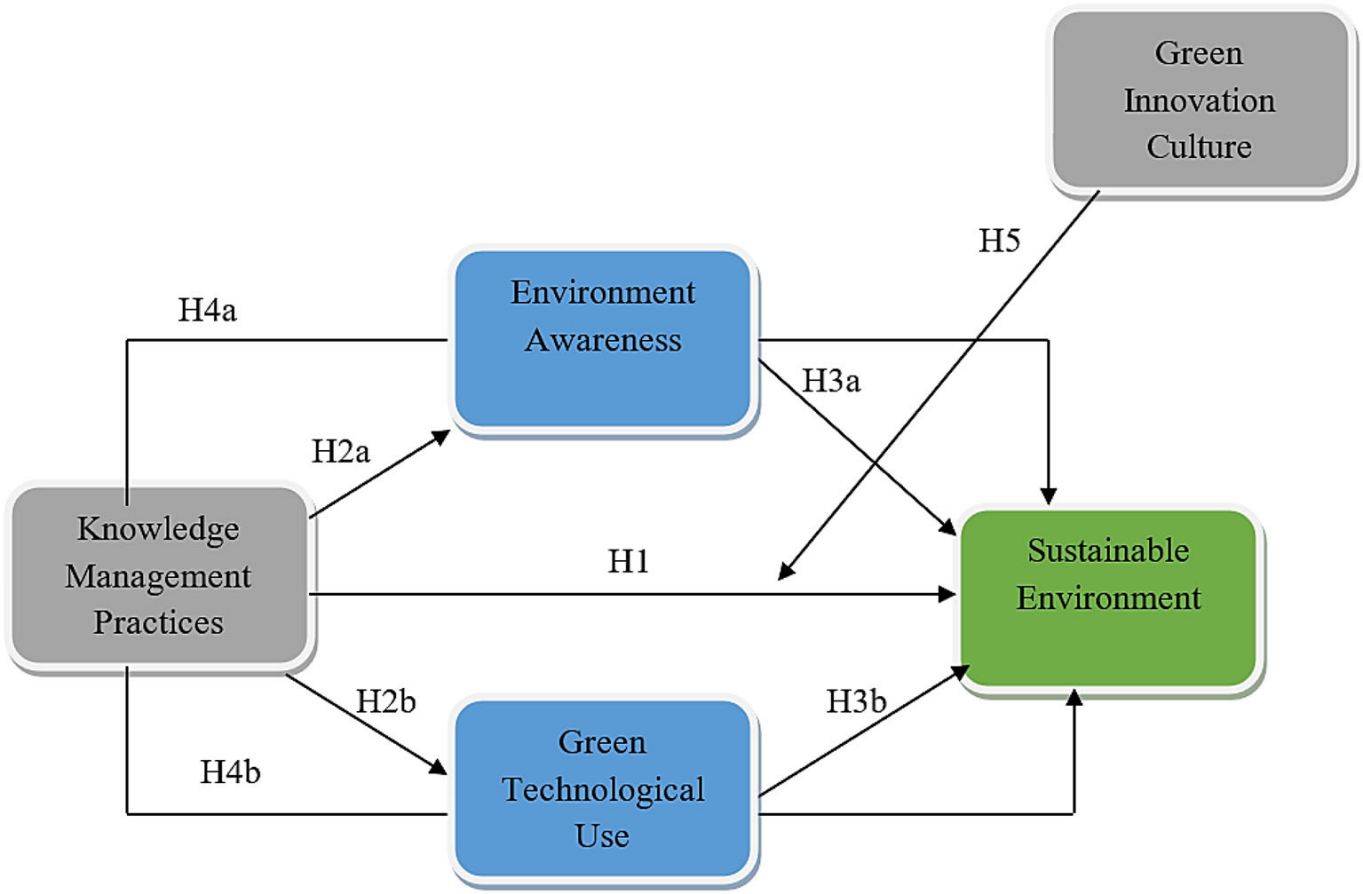
Theoretical framework.

Based on the literature support and hypothesis development, a framework (Figure 1) has been developed as mentioned below.

## Methodology

Quantitative research design along with the deductive approach was used in this study to analyze the hypotheses. The hypotheses helped the researcher to determine the impact on dependent variables because of the independent variables. Quantitative research design enabled the elimination of biases in this study so that the results are reliable. A self-administered survey was deployed for data collection (Prati et al., 2010). The questionnaire was kept clear and precise to obtain data rationality. Moreover, the participants were told that there were no right or wrong answers and the participants were to be as natural as possible. A total of 400 questionnaires were distributed to the participants. After two to three visits and reminders, 378 questionnaires were obtained (see Table 1). Approximately 2 weeks were spent obtaining the responses. The questionnaires were collected from the study participants and were then screened. A total of 22 questionnaires were discarded as the responses in those questionnaires were either improper or incomplete. Thus, the useable response rate was 94.5%. The responses were then scrutinized and examined using statistical software Smart-PLS. The data were collected from the managerial level personnel of the construction industry; therefore, they were the target population for the study.

**Table 1:** Demographics analysis.

**Demographics**	**Frequency**	**Percentage**
**Gender**		
Male	216	57.14%
Female	162	42.86%
**Age (years)**		
20–30	84	22.22%
31–40	163	43.12%
41–50	75	19.84%
Above 50	56	14.81%
**Education**		
Bachelors	68	17.99%
Masters	201	53.17%
Ph.D. and others	109	28.84%
**Organizational tenure (years)**		
Less than 1	86	22.75%
1–3	127	33.60%
4–6	119	31.48%
More than 6	46	12.1%

*N = 378*.

The sample from the entire population under study was selected using convenience sampling technique. According to Etikan et al. (2015), this sampling technique enabled the researcher to collect the data from the readily available respondents in a short time span and in a less expensive way. A sample size of 378 was determined for the current study. The managerial level personnel of the construction industry of China were the unit of analysis for this study.

### Statistical Tool

Smart-PLS 3.3.3 software was used to examine structure equation technique (SEM) that was required for this study. According to Henseler et al. (2015), this software helps to provide thorough analysis of small data by developing path model in a short span of time. The software uses a measurement model and structural model to examine the data. In the measurement model, data validity and reliability are determined, while in the structural model, the hypotheses of the study are tested. The *p*-values and *t*-statistics helps to determine whether the hypothesis is accepted or not.

### Measurement

The data for each item of the construct was obtained with the help of a 5-point Likert scale. The measurement for this study is as follows.

#### Knowledge Management Practices

The scale for KM practices comprised of 11 items was adopted from Bennett and Gabriel (1999).

#### Sustainable Environment

The scale for sustainable environment comprised of 4 items was adopted from Calik and Bardudeen (2016).

#### Environment Awareness

The scale for environment awareness comprised of 4 items was adopted from Cao and Chen (2019).

#### Green Technological Use

The scale for green technological use comprising 6 items was adopted from Luu (2021).

#### Green Innovative Culture

The scale for green innovative culture comprising 6 items was adopted from Wong et al. (2016).

### Demographic Details

The participation of males and females in the current study was 57.14 and 42.86%, respectively. A total of 22.22% of the sample was aged between 20 and 30 years, 43.12% were between the age of 31 and 40 years, the participation of managers between the age of 41–50 years was 19.84%, while 14.81% were above 50 years. Moreover, 17.99% of the participants held a bachelor's degree, 53.17% held a master's degree, and 28.84% held a Ph.D. or other degree. Furthermore, the managers who had an organizational tenure of <1 year were 22.75, 33.60% had 1–3 years of organizational tenure, 31.48% had 4–6 years of organizational tenure, and 12.10% had more than 6 years of organizational tenure.

## Data Analysis and Results

### Measurement Model

**Figure 2:**
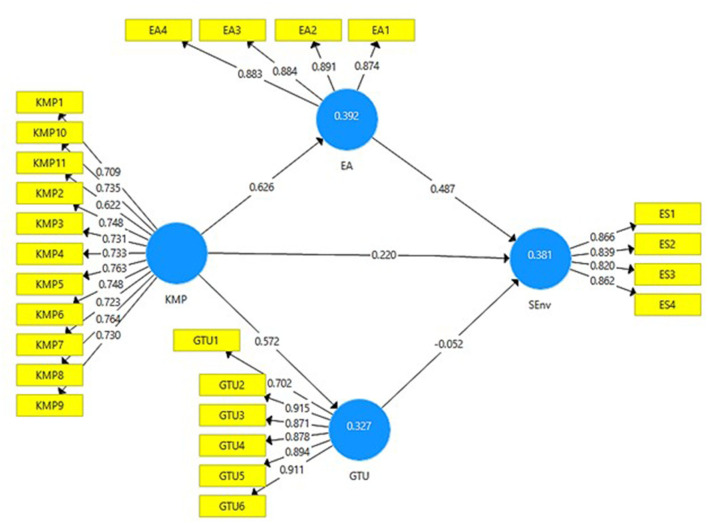
Output of measurement model without moderation. KMP, Knowledge Management Practices; EA, Environmental Awareness; GTU, Green Technological Use; SEnv, Sustainable Environment.

**Figure 3:**
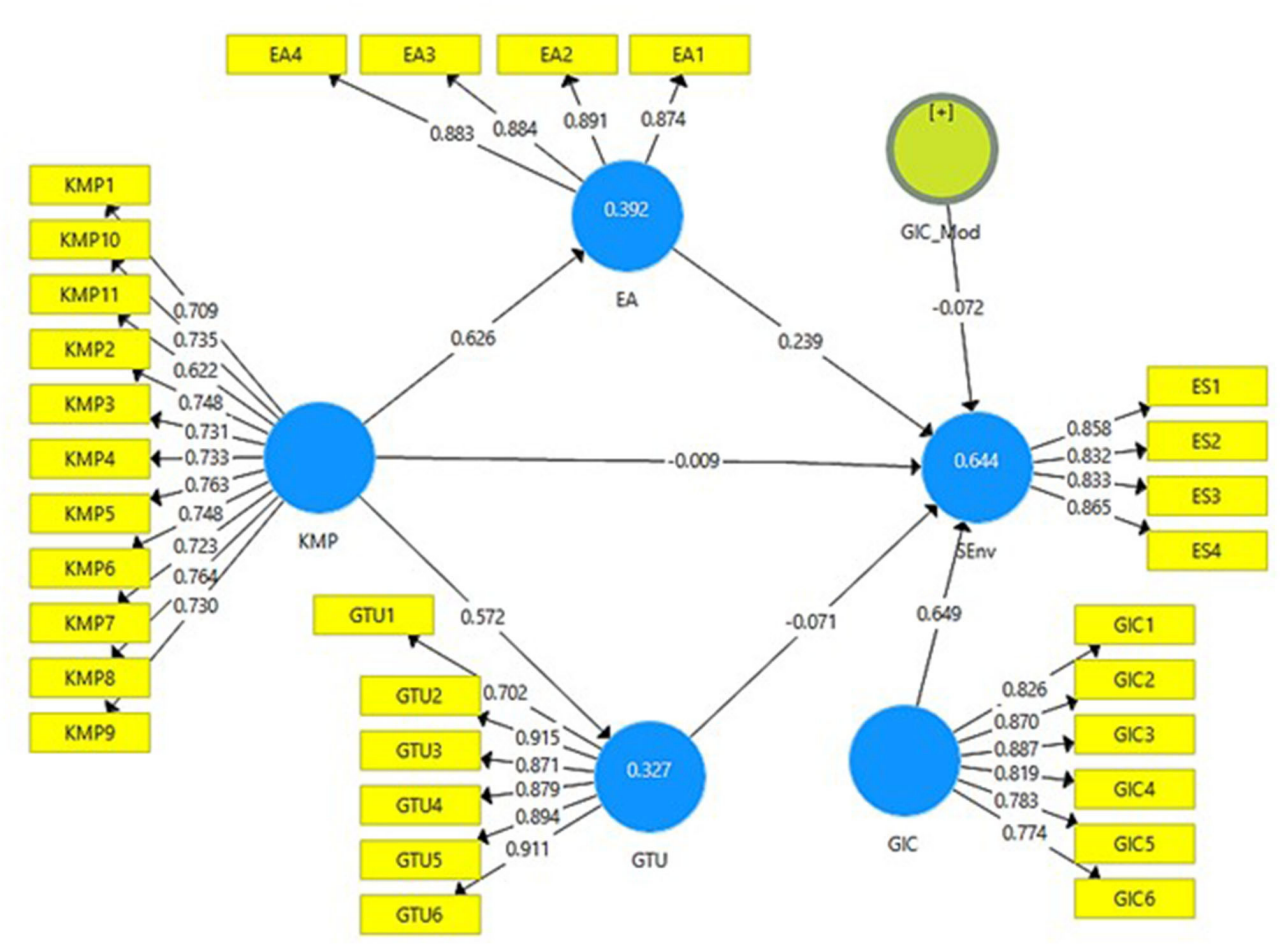
Output of measurement model with moderation. KMP, Knowledge Management Practices; EA, Environmental Awareness; GTU, Green Technological Use; SEnv, Sustainable Environment; GIC, Green Innovative Culture.

Figures 2, 3 below presents the output of measurement model without moderation and with moderation. It can be seen how much the independent variables contribute to the outcome variables of this study.

Table 2 demonstrates the factor loadings and variance inflation factor (VIF) of each item of KM practices, sustainable environment, environment awareness, and green technological use. According to Huo et al. (2020), the value of factor loadings for each item should be higher than 0.60. The factor loadings for the present ranged from 0.622 to 0.911, therefore, the obtained values are fair. The collinearity issue in the model is determined through VIF. The value of outer VIF must be lower than 5 (Hair et al., 2017). The result of VIF for the study indicates that no collinearity issue in the model was present as the value of VIF ranged from 1.474 to 4.294. Model assessment (direct model) also shows the construct reliability and validity using Cronbach alpha (α), composite reliability, and AVE. Reliability is said to be satisfactory if the value of Cronbach alpha is higher than 0.70 (Henseler et al., 2015), and the composite reliability must also be higher than 0.70 (Peterson and Kim, 2013). The Cronbach alpha values and composite reliability for the variables under study were more than 0.70, which suggests that the data was reliable. Moreover, the AVE values must be above 0.60 (Kim and Shim, 2018). The AVE value for this study above the threshold level, indicating the presence of convergent validity.

**Table 2:** Model assessment (direct model).

	**Factor loadings**		**VIF**	**Construct reliability and validity**		
				**α**	**Composite Reliability**	**AVE**
Knowledge management practices	KMP1	0.709	2.067			
	KMP2	0.748	2.869			
	KMP3	0.731	2.421			
	KMP4	0.733	1.817			
	KMP5	0.763	2.980	0.912	0.925	0.531
	KMP6	0.748	3.138			
	KMP7	0.723	2.621			
	KMP8	0.764	3.684			
	KMP9	0.730	2.103			
	KMP10	0.735	2.125			
	KMP11	0.622	1.546			
Environment awareness	EA1	0.874	2.558			
	EA2	0.891	2.783	0.906	0.934	0.780
	EA3	0.884	2.579			
	EA4	0.883	2.646			
Green technological use	GTU1	0.702	1.474			
	GTU2	0.915	4.375			
	GTU3	0.871	4.294	0.931	0.946	0.748
	GTU4	0.878	3.969			
	GTU5	0.894	4.081			
	GTU6	0.911	3.760			
Sustainable environment	SEnv1	0.866	2.203			
	SEnv2	0.839	2.095	0.869	0.910	0.718
	SEnv3	0.820	2.017			
	SEnv4	0.862	2.225			

*KMP, Knowledge Management Practices; EA, Environmental Awareness; GTU, Green Technological Use; SEnv, Sustainable Environment; GIC, Green Innovative Culture; VIF, Variance Inflation Factor; α, Cronbach Alpha; AVE, Average Variance Extracted*.

Heterotrait-Monotrait (HTMT) ratio and Fornell and Larker Criteria are two tests that determine the discriminant validity of the data (see Table 3). Discriminant validity explains whether one variable is different from the rest of the variables. The value of HTMT ratio below 0.90 tells that discriminant validity exists. The results obtained showed that HTMT ratio for each construct was below 0.90 (ranged from 0.376 to 0.673), therefore, discriminant validity exists between the variables. Similarly, considering the Fornell and Larker Criteria the value on the top of every column should be more than the following values (Franke and Sarstedt, 2019). The table demonstrates that discriminant validity exists (considering the Fornell and Larker Criterion) as the criteria for this test has been met.

**Table 3:** Discriminant validity.

**Fornell–Larcker criterion**					**Heterotrait–Monotrait ratio**				
**Constructs**	**EA**	**GTU**	**KMP**	**SEnv**	**Constructs**	**EA**	**GTU**	**KMP**	**SEnv**
EA	0.883				EA				
GTU	0.558	0.865			GTU	0.601			
KMP	0.626	0.572	0.729		KMP	0.673	0.610		
SEnv	0.596	0.345	0.495	0.847	SEnv	0.665	0.376	0.546	

*N = 378. KMP, Knowledge Management Practices; EA, Environmental Awareness; GTU, Green Technological Use; SEnv, Sustainable Environment*.

Table 4 shows the values of *R*-square for environmental awareness, green technological use, and sustainable environment. The value of *R*^2^ for environmental awareness, green technological use, and sustainable environment are 0.392, 0.327, and 0.381, respectively. This suggests that the model is substantial and good.

**Table 4:** R-square values for the variables.

	***R*-square**
EA	0.392
GTU	0.327
SEnv	0.381

*N = 378. EA, Environmental Awareness; GTU, Green Technological Use; SEnv, Sustainable Environment*.

The collinearity issue in the model is determined through VIF. The value of inner VIF must be lower than 5 (Syazwan Wahab et al., 2017). The result of inner VIF for the study (see Table 5) indicates that no collinearity issue in the model was present as the value of inner VIF ranged from 1.000 to 2.231.

**Table 5:** Collinearity statistics (inner-VIF values).

	**EA**	**GIC**	**GTU**	**KMP**	**Moderating effect 1**	**SEnv**
EA						2.051
GIC						1.594
GTU						1.670
KMP	1.000		1.000			2.231
Moderating effect 1						1.433
SEnv						

*N = 378. VIF, Variation Inflation Factor; KMP, Knowledge Management Practices; EA, Environmental Awareness; GTU, Green Technological Use; SEnv, Sustainable Environment*.

### Structural Model

**Figure 4:**
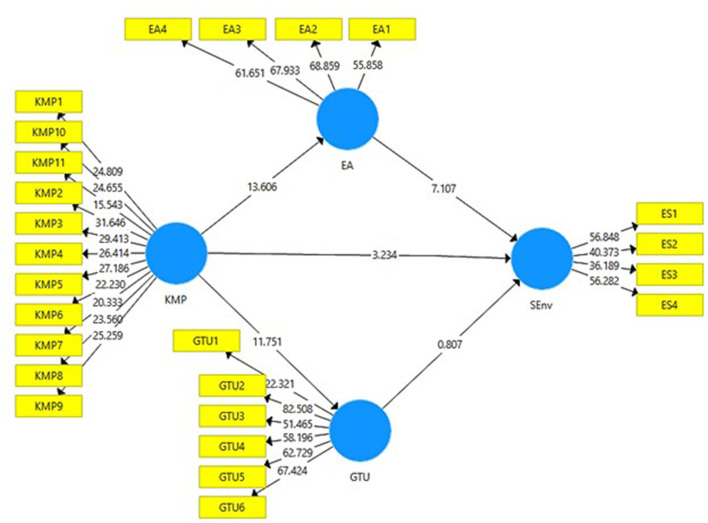
Structural model bootstrapping without moderation. KMP, Knowledge Management Practices; EA, Environmental Awareness; GTU, Green Technological Use; SEnv, Sustainable Environment.

Figure 4 presents the output of structural model bootstrapping without moderation which includes values for *t*-statistics. The acceptance or rejection of the study hypotheses is determined through PLS-SEM bootstrapping model. A total of 95% corrected bootstrap is considered to examine the hypotheses of the study.

The direct effect, indirect effect, and moderating effect can be seen in Tables 6, 7, **9**. The hypotheses are either accepted or rejected based on *t*-statistics and *p*-values. The value of *t*-statistics must be above 1.96 (Johnson, 2019). Significance value or *p*-value should be lower than 0.05 (Di Leo and Sardanelli, 2020). Moreover, the table demonstrates the effect size (*f*^2^) or strength of the model. Higher strength is indicated by the values near 1 and lower strength is indicated by the values near 0 (McKnight et al., 2002).

**Table 6:** Direct effects of the variable.

**Paths**	**H**	**O**	**M**	**SD**	**T-statistics**	**Effect size (*f*^2^)**	***P*-value**	**Results**
KMP → SEnv	H_1_	0.220	0.228	0.066	3.305	0.042	0.001^***^	Accepted
KMP → EA	H_2a_	0.626	0.630	0.044	14.282	0.645	0.000^**^	Accepted
KMP → GTU	H_2b_	0.572	0.577	0.049	11.688	0.486	0.000^**^	Accepted
EA → SEnv	H_3a_	0.487	0.487	0.067	7.318	0.210	0.000^***^	Accepted
GTU → SEnv	H_3b_	−0.052	−0.057	0.069	0.762	0.003	0.446	Rejected

*N = 378*,

*** *p < 0.001*,

** *p < 0.005*.

*H, Hypothesis; O, Original Sample; M, Sample Mean; SD, Standard Deviation; SRMR = 0.098; NFI = 0.747; KMP, Knowledge Management Practices; EA, Environmental Awareness; GTU, Green Technological Use; SEnv, Sustainable Environment*.

**Table 7:** Indirect effects of the variable.

**Paths**	**H**	**O**	**M**	**SD**	**t-statistics**	***P*-value**	**Results**
KMP → EA → SEnv	H_4a_	0.305	0.306	0.042	7.237	0.000^***^	Accepted
KMP → GTU → SEnv	H_4b_	−0.030	−0.033	0.040	0.748	0.455	Rejected

*N = 378*,

*** *p < 0.001*.

*H, Hypothesis; O, Original Sample; M, Sample Mean; SD, Standard Deviation; KMP, Knowledge Management Practices; EA, Environmental Awareness; GTU, Green Technological Use; SEnv, Sustainable Environment*.

Table 6 shows H1, H2a, H2b, H3a, and H3b. H1 states that there is a relationship between KM practice on sustainable environment and this hypothesis was accepted as (*t* = 3.305, *p* < 0.05). The model has a very low strength as (*f*^2^ = 0.042). H2a was also accepted as *t* value was more than 1.96 and *p*-value was <0.05 (*t* = 14.282, *p* = 0.000), thus, there is a relationship between KM practice on environmental awareness. The model has a medium-to-high strength as (*f*^2^ = 0.645). H2b proposed that there is a relationship between knowledge management practice on green technological usage. The result for this hypothesis was (*t* = 11.688, *p* = 0.000), which indicates the acceptance of this hypothesis. The model has a medium strength as (*f*^2^ = 0.486). H3a was accepted as (*t* = 7.318, *p* = 0.000) indicating that there is a relationship between environmental awareness on practice on sustainable environment. The model has a low strength as (*f*^2^ = 0.210). The result for H3b showed that *t* < 1.96 and *p* > 0.05), which indicates that there is no relationship between green technological usage on sustainable environment, thus H3b hypothesis was rejected. The model has a very low strength as (*f*^2^ = 0.003).

The value of Normed Fixed Index (NFI) determines the model fitness. This value must be between 1 and 0 (Elsayed and Aneis, 2021). The value of NFI came out to be 0.747 which indicates that the model fitness is high.

H4a has been accepted as *t* = 7.237 and *p* = 0.000 indicating that environmental awareness mediates the relationship between knowledge management practice on sustainable environment. Moreover, H4b proposed that green technological usage mediates the relationship between knowledge management practice on sustainable environment. The result for this hypothesis was (*t* = 0.748, *p* = 0.455) which indicates the rejected of H4b hypothesis (see Table 7).

**Figure 5:**
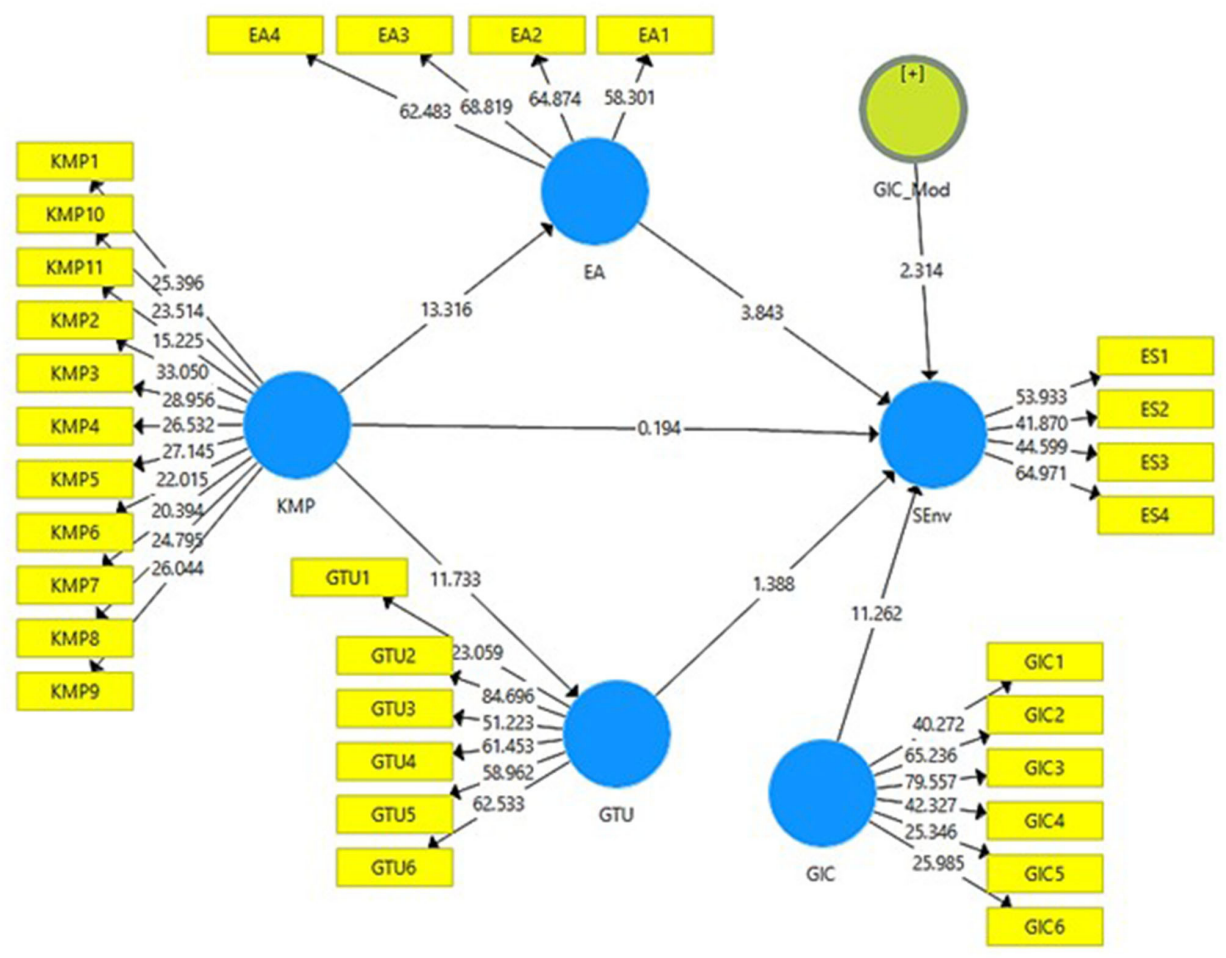
Structural model bootstrapping with moderation. KMP, Knowledge Management Practices; EA, Environmental Awareness; GTU, Green Technological Use; SEnv, Sustainable Environment; GIC, Green Innovative Culture.

The validation of the data (reliability and validity) was again conducted with the moderating variable (i.e., green innovative culture) in the relationship between knowledge management practices and sustainable environment. Table 8 shows that the values of factor loadings, VIF, Cronbach alpha, composite reliability, and AVE were above their threshold level and Table 9 and Figure 5 shows the moderating effects of variables.

**Table 8:** Model assessment (moderation).

				**Construct reliability and validity**		
	**Factor loadings**		**VIF**	**α**	**Composite reliability**	**AVE**
Knowledge management practices	KMP1	0.709	2.067			
	KMP2	0.748	2.869			
	KMP3	0.731	2.421			
	KMP4	0.733	1.817			
	KMP5	0.763	2.980	0.912	0.925	0.531
	KMP6	0.748	3.138			
	KMP7	0.723	2.621			
	KMP8	0.764	3.684			
	KMP9	0.730	2.103			
	KMP10	0.735	2.125			
	KMP11	0.622	1.546			
Environment awareness	EA1	0.874	2.558			
	EA2	0.891	2.783	0.906	0.934	0.780
	EA3	0.884	2.579			
	EA4	0.883	2.646			
Green technological use	GTU1	0.702	1.474			
	GTU2	0.915	4.375			
	GTU3	0.871	4.294	0.931	0.946	0.748
	GTU4	0.878	3.969			
	GTU5	0.894	4.081			
	GTU6	0.911	3.760			
Sustainable environment	SEnv1	0.866	2.203			
	SEnv2	0.839	2.095	0.869	0.910	0.718
	SEnv3	0.820	2.017			
	SEnv4	0.862	2.225			
Green innovative culture	GIC1	0.826	2.752			
	GIC2	0.870	3.090			
	GIC3	0.887	3.743	0.928	0.982	0.718
	GIC4	0.819	2.530			
	GIC5	0.783	2.314			
	GIC6	0.774	2.474			

*KMP, Knowledge Management Practices; EA, Environmental Awareness; GTU, Green Technological Use; SEnv, Sustainable Environment; GIC, Green Innovative Culture; VIF, Variance Inflation Factor; α, Cronbach*.

**Table 9:** Moderating effects of the variable.

**Paths**	**H**	**O**	**M**	**SD**	**t-statistics**	***P*-value**	**Results**
KMP × GIC → SEnv	H_5_	−0.072	−0.076	0.030	2.386	0.017^*^	Accepted

*N = 378*,

* *p < 0.05. H, Hypothesis; O, Original Sample; M, Sample Mean; SD, Standard Deviation; KMP, Knowledge Management Practices; GIC, Green Innovative Culture; SEnv, Sustainable Environment*.

H5 was also accepted as *t* value was more than 1.96 and *p* value was <0.05 (*t* = 2.386, *p* = 0.017), thus, green innovative culture moderates the relationship between knowledge management practices and sustainable environment (see Table 9).

## Discussion

Because of the growing environmental awareness and the subsequent consequences created by its own mismanagement, a sustainable environment has become a requirement and has been highlighted greatly (Lin and Niu, 2018). The goal of this study was to evaluate the function of knowledge management techniques in a sustainable environment, as well as the role of environmental awareness and green technological use as mediators. The study also looked at the function of green innovative culture in moderating the relationship between knowledge management practices and environmental sustainability. The direct relationship of knowledge management practices with sustainable environment proved to be significant, indicating that if knowledge among the stakeholders is properly managed and disseminated then it becomes a beneficial component in achieving sustainable environment for organizations.

It is also due to the fact that organizations all across the globe acknowledge knowledge as a main source of competitive advantage and value development (Castellani et al., 2022). It is also supported by some researchers who considered that proper management of knowledge is critical for the viability of sustainable development (Zhang et al., 2022). The relationships of knowledge management practices with environmental awareness and green technological usage also proved significance indicating the importance of knowledge management across the globe. These results proved that proper knowledge management could lead proper awareness about the environment which is a global issue and helps in understanding the use of green technologies. If information is adequately transferred or managed among stakeholders, then it would also be beneficial in structuring sustainable environments at organizational level. Similar sort of relationships are also supported by some previous scholars of knowledge management (Polas et al., 2021).

Knowledge management practices in the past have proved their significance on impacting GIs which is a component of green technologies (Wang H. et al., 2021). The next components of the current research were to evaluate the impact of environmental awareness and green technological use on achieving sustainable environments. The results proved that both were directly related to sustainable environments. In the past, no research was conducted in this regard to evaluate the direct impact of environmental awareness on sustainable environment and green technological usage on sustainable environment. These results are obtained possibly due to the fact that if awareness about green technologies and practices is properly dispersed among the stakeholders then it surely would have positive effects on the environment. Previous research has shown that being aware of green practices can lead to a favorable attitude about implementing them (Sadiq et al., 2022).

People who are aware of the possible consequences of non-sustainable activities such as improper disposal of plastic and other wastes, for example, might create a strong sense of self-belief and moral duty to take necessary action to reduce such practices (Ojo and Fauzi, 2020). Therefore, it could lead to achieving a sustainable environment. Similarly, the concept of green technology is a subset of green technologies that is used to safeguard the environment (Fernando et al., 2016). Hence, it also impacted the sustainability of the environment positively. The mediating effects of environmental awareness and green technological usage were also evaluated in this research which indicated that direct relationship of knowledge management practices with the help of environmental awareness would be enhanced toward attaining sustainable environments. This was possible because if knowledge is managed effectively, then it improves awareness about the environment among the stakeholders which leads to achieving a sustainable environment.

Some similar results in different perspectives were also obtained in which growing environmental awareness, along with concerns about safe foods, has prompted challenges to current agricultural techniques in order to ensure the environment's long-term sustainability (Bertola et al., 2021). Similarly, the mediating role of green technological usage could have produced good results but in this current research their mediating roles were non-significant indicating that if a direct relationship of knowledge management practices is significant toward sustainable environment, then there remains no need for green technological usage for enhancing this direct relationship. This relationship is itself a strong association. The idea was generated on the basis of a significant mediating role of green information technology between green university and sustainable development of environment (Alipour et al., 2019).

The moderator effects of green innovative culture were also evaluated in this research. The results were similar to what was expected as it significantly regulated the relationship of knowledge management practices with sustainable environment. These results were supported by the possible reasoning that researchers in the environmental business marketing paid special attention to GI (Yousaf, 2021). Green technology helped businesses create a competitive advantage while simultaneously increasing environmental sustainability (Zameer et al., 2020). The results proved that the culture of GI regulates the functioning of knowledge management and sustainability accomplishment in environments.

## Practical Implications, Limitations and Future Directions, and Conclusion

### Practical Implications

Knowledge management practices ought to enhance environmental awareness and the sustainable environment within the construction sector, therefore, the management of different companies must encourage knowledge management practices through leading by example, providing incentives to employees, or fostering the right mindset among employees. Moreover, management must devise policies to promote a green innovative culture within the organization so that a sustainable environment can be developed. A green innovative culture can also be developed with the help of a recycling program, and this should be specifically dedicated to promoting recycling. Furthermore, environmental awareness must be created among the managers by providing orientations and training to the managers and assigning socially responsible tasks to employees considering the green practices and procedures for promoting the intelligent use of information and resources available. The managers can then encourage their employees to engage in activities leading to environmental protection.

### Limitations and Future Directions

Like other studies, this study also has some limitations. This study examined the role of knowledge management practices on a sustainable environment with the mediating role of environmental awareness and green technological use and the moderating role of green innovative culture, so future studies can investigate other mediating variables such as green supply chain adoption and green process design and moderating variable such as environmental consciousness. The sample size of this study was small; thus, future studies can use a larger sample size for generalizability of the data. Moreover, this study used a cross-sectional study design, so future studies can use a longitudinal study design. Furthermore, qualitative techniques can also be applied in future studies in order to examine the research model.

### Conclusion

The need for sustainable environment has been increasing because of an increase in environmental awareness and consequences of environmental problems. Moreover, a sustainable environment can be developed through knowledge management practices, environmental awareness, and green technological use. Therefore, this study investigated the role of knowledge management practices on sustainable environment with the mediating role of environmental awareness and green technological use and the moderating role of green innovative culture. The study was conducted on managerial level personnel in the construction industry in China. The study found that knowledge management practice has a relationship with sustainable environment, environmental awareness, and green technological use. Also, environment awareness has a relationship with sustainable environment. However, no relationship was found between green technological use and sustainable environment. Moreover, it was found in the study that environmental awareness mediated the relationship between knowledge management practices and sustainable environment, but green technological use did not mediate the relationship between knowledge management practices and sustainable environment. Furthermore, green innovative culture moderated the relationship between knowledge management practices and sustainable environment.

## Data Availability Statement

The original contributions presented in the study are included in the article/supplementary material, further inquiries can be directed to the corresponding author/s.

## Author Contributions

YY conceived and designed the concept. AW collected the data and wrote the paper. All authors read and agreed to the published version of the manuscript.

## Funding

This work was funded within the project No. (2019ZDWT14) entitled: Investigation and Research on the construction of Shaanxi new era civilization Practice Center, supported by Shaanxi Social Science Foundation.

## Conflict of Interest

The authors declare that the research was conducted in the absence of any commercial or financial relationships that could be construed as a potential conflict of interest.

## Publisher's Note

All claims expressed in this article are solely those of the authors and do not necessarily represent those of their affiliated organizations, or those of the publisher, the editors and the reviewers. Any product that may be evaluated in this article, or claim that may be made by its manufacturer, is not guaranteed or endorsed by the publisher.
